# Podocalyxin-like protein as a predictive biomarker for benefit of neoadjuvant chemotherapy in resectable gastric and esophageal adenocarcinoma

**DOI:** 10.1186/s12967-018-1668-3

**Published:** 2018-10-24

**Authors:** David Borg, Anna H. Larsson, Charlotta Hedner, Björn Nodin, Anders Johnsson, Karin Jirström

**Affiliations:** Department of Clinical Sciences Lund, Division of Oncology and Pathology, Lund University, Skåne University Hospital, 221 85 Lund, Sweden

**Keywords:** Esophageal adenocarcinoma, Gastric adenocarcinoma, Neoadjuvant chemotherapy, PODXL

## Abstract

**Background:**

We have previously shown that podocalyxin-like protein (PODXL) is a prognostic biomarker for poor survival in gastric and esophageal adenocarcinoma treated with surgery up-front. The aim of the present study was to assess PODXL expression in tumors from patients treated with neoadjuvant ± adjuvant (i.e. preoperative with or without postoperative) chemotherapy, with regard to histopathologic response, time to recurrence (TTR) and overall survival (OS).

**Methods:**

The neoadjuvant cohort encompasses 148 consecutive patients who received neoadjuvant ± adjuvant chemotherapy for resectable gastric or esophageal adenocarcinoma between 2008 and 2014. Immunohistochemical expression of PODXL was assessed in pre-neoadjuvant biopsies, resected primary tumors and lymph node metastases. Histopathologic response was evaluated using the Chirieac grading. TTR and OS were estimated using Kaplan–Meier and Cox regression analyses. To investigate a potential predictive role for PODXL, the neoadjuvant cohort was pooled with the previously reported surgery up-front cohort.

**Results:**

The majority (> 95%) of the patients were treated with fluoropyrimidine- and oxaliplatin-based chemotherapy. Patients with high PODXL expression in their pre-neoadjuvant biopsies had a superior histopathologic response (notably 36% with no residual cancer cells) compared to those with negative or low PODXL expression, and were all recurrence-free at last follow-up. In the pooled cohort, no benefit of chemotherapy could be shown for PODXL negative cases, whereas PODXL positive (low or high) cases had a prolonged TTR and OS when treated with neoadjuvant ± adjuvant chemotherapy compared to surgery alone. The potential predictive role of PODXL was further strengthened for TTR in Cox regression analyses, especially for patients treated with neoadjuvant fluoropyrimidine and oxaliplatin for a minimum of 8 weeks, with a significant interaction term in both unadjusted (p = 0.006) and adjusted (p = 0.024) analyses. The interaction term was not statistically significant for overall survival.

**Conclusions:**

Patients with resectable gastric or esophageal adenocarcinoma with high PODXL expression in their diagnostic biopsies have an excellent prognosis when treated with neoadjuvant ± adjuvant fluoropyrimidine- and oxaliplatin-based chemotherapy. If the suggested predictive role of PODXL for benefit of chemotherapy can be confirmed, patients with PODXL negative tumors could be spared chemotherapy and treated with surgery alone.

**Electronic supplementary material:**

The online version of this article (10.1186/s12967-018-1668-3) contains supplementary material, which is available to authorized users.

## Background

Despite declining incidence, gastric adenocarcinoma remains the third most common cause of cancer death worldwide [1]. The incidence of esophageal adenocarcinoma has dramatically increased in the last four decades, and in several Western countries it is now more common than squamous cell carcinoma, which is the predominant subtype in Asia and most developing countries [2].

For fit patients with localized gastric or esophageal adenocarcinoma without distant metastasis (M0) the mainstay of curative treatment is surgical resection. However, since merely 20–25% of resected patients achieve long-term survival, additional treatment with chemo- or chemoradiotherapy has emerged and been shown to improve survival. One of the standard treatment strategies, particularly in Europe [3, 4], is perioperative (i.e. neoadjuvant + adjuvant) chemotherapy based on the MAGIC trial [5] and FFCD 9703 trial [6], where the 5-year survival rate increased with 13–14% compared to surgery alone. The established chemotherapy backbone is a combination of fluoropyrimidine (fluorouracil or capecitabine) and platinum (cisplatin or oxaliplatin) with or without a third drug (epirubicin or docetaxel). Although long-term survival is yet to be reported, preliminary survival data from the FLOT4-AIO trial [7] indicate that the FLOT regimen with fluorouracil, oxaliplatin and docetaxel will become the new reference regimen.

Since only a minority of the patients actually benefit from perioperative chemotherapy it would be of great importance to identify these patients in advance so that the rest can be spared the burden of an unnecessary and toxic treatment. However, no predictive tools for such decision making have yet been established.

Podocalyxin-like protein (PODXL) is a cell surface transmembrane glycoprotein encoded on chromosome 7q32-q33 and a member of the CD34-family. It was initially identified in glomerular podocytes as an anti-adhesive protein [8], but further studies have shown that PODXL is involved in several physiologic processes such as regulating vascular permeability [9], leucocyte-endothelial cell interaction [10, 11], hematopoiesis [12] and neural development [13].

High expression of PODXL has been linked to poor prognosis in a wide range of malignancies including gastrointestinal adenocarcinomas such as colorectal cancer [14–16], pancreatic and periampullary cancer [17–20] and gastric cancer [21]. Proposed mechanisms for PODXL-associated tumorigenesis are increased tumor cell migration, invasion and metastatic potential [22–24], possibly by inducing epithelial-mesenchymal transition [25, 26]. Other suggested mechanisms are immune evasion [27] or stabilization of glucose transporters [28].

We have previously shown that PODXL is an independent prognostic biomarker for poor survival in a cohort of resected gastric and esophageal adenocarcinomas treated with surgery up-front [29]. The aim of the present study was to assess PODXL status in a more recent cohort, treated with neoadjuvant ± adjuvant chemotherapy, with regard to histopathologic response, time to recurrence (TTR) and overall survival (OS). Furthermore, we also wanted to determine if PODXL may be a predictive biomarker for benefit of neoadjuvant ± adjuvant chemotherapy.

## Methods

### Study design and participants

The neoadjuvant cohort consists of 148 consecutive patients diagnosed with resectable gastric or esophageal adenocarcinoma who received neoadjuvant ± adjuvant chemotherapy at the Skåne University Hospital in Lund and Malmö between January 1, 2008 and December 31, 2014. This cohort has been described previously [30] but for the present study, patients treated with neoadjuvant chemoradiotherapy or with palliative chemotherapy followed by salvage surgery were excluded. Prior to start of neoadjuvant treatment, all patients were discussed at a multidisciplinary tumor board where clinical stage was determined based on findings on endoscopy and computerized tomography. Diagnostic laparoscopy was not part of routine work-up unless peritoneal carcinomatosis was suspected. Data on recurrence and vital status were updated until December 31, 2017. Patient and tumor characteristics in the neoadjuvant cohort are described in Table 1 and treatment data in Additional file 1. The vast majority (> 95%) of the patients were treated with fluoropyrimidine- and oxaliplatin-based chemotherapy (EOX, FOLFOX or FLOX).

**Table 1:** Patient and tumor characteristics in the neoadjuvant cohort

	Patients n (%)
N	148
Age (years)	
Mean	63.2
Median	65.2
Range	21.1–81.0
Sex	
Female	58 (39.2)
Male	90 (60.8)
Location	
Esophagus	62 (41.9)
Stomach	86 (58.1)
cT stage	
T1	1 (0.7)
T2	54 (36.5)
T3	87 (58.8)
T4	6 (4.1)
cN stage	
N0	80 (54.1)
N1	50 (33.8)
N2	14 (9.5)
N3	4 (2.7)
cM stage	
M0	136 (91.9)
M1	12 (8.1)
Lymph node metastasis in M1-position	6
Liver metastasis	3
Adrenal gland metastasis	1
Ovarian metastasis	1
Ascites	1
Neoadjuvant treatment	
Chemotherapy NOS	148
Fluoropyrimidine + platinum ≥ 8 weeks, no irinotecan	121 (81.8)
Resection	
Yes	118 (79.7)
No	30 (20.3)
Peroperative findings of advanced disease	20
Liver metastasis found after one cycle of chemotherapy	1
Progressive disease	1
Deteriorated performance status	3
Death	2
Patient’s wish	3
Adjuvant treatment	
Chemotherapy NOS	78 (66.7)
Fluoropyrimidine + platinum ≥ 8 weeks, no irinotecan	46
Chemoradiotherapy	10 (8.5)
None	29 (24.8)
Missing data	1
No surgery	30
Histopathologic response	
0% residual cancer cells	13 (11.1)
1–10%	13 (11.1)
11–50%	46 (39.3)
> 50%	45 (38.5)
Missing data	1
No surgery	30
ypT stage	
T0	13 (11.1)
T1	14 (12.0)
T2	22 (18.8)
T3	42 (35.9)
T4	26 (22.2)
Missing data	1
No surgery	30
ypN stage	
N0	52 (44.1)
N1	28 (23.7)
N2	18 (15.3)
N3	20 (16.9)
No surgery	30
ypM stage	
M0	113 (95.8)
M1	5 (4.2)
Lymph node metastasis in M1-position	1
Liver metastasis	1
Ovarian metastasis	1
Peritoneal deposit	2
No surgery	30
Residual tumor status	
R0	97 (82.2)
R1	19 (16.1)
R2	2 (1.7)
No surgery	30
Number of examined lymph nodes	
Mean	36
Median	35
Range	4–88
Differentiation grade	
Low grade	3 (2.4)
Intermediate grade	52 (42.3)
High grade	68 (55.3)
Missing data	25
Lauren classification	
Intestinal	65 (52.8)
Mixed	16 (13.0)
Diffuse	42 (34.1)
Missing data	25
Follow-up resected patients (years)	
Median (95% CI)	5.8 (5.2–6.4)

**Fig. 1:**
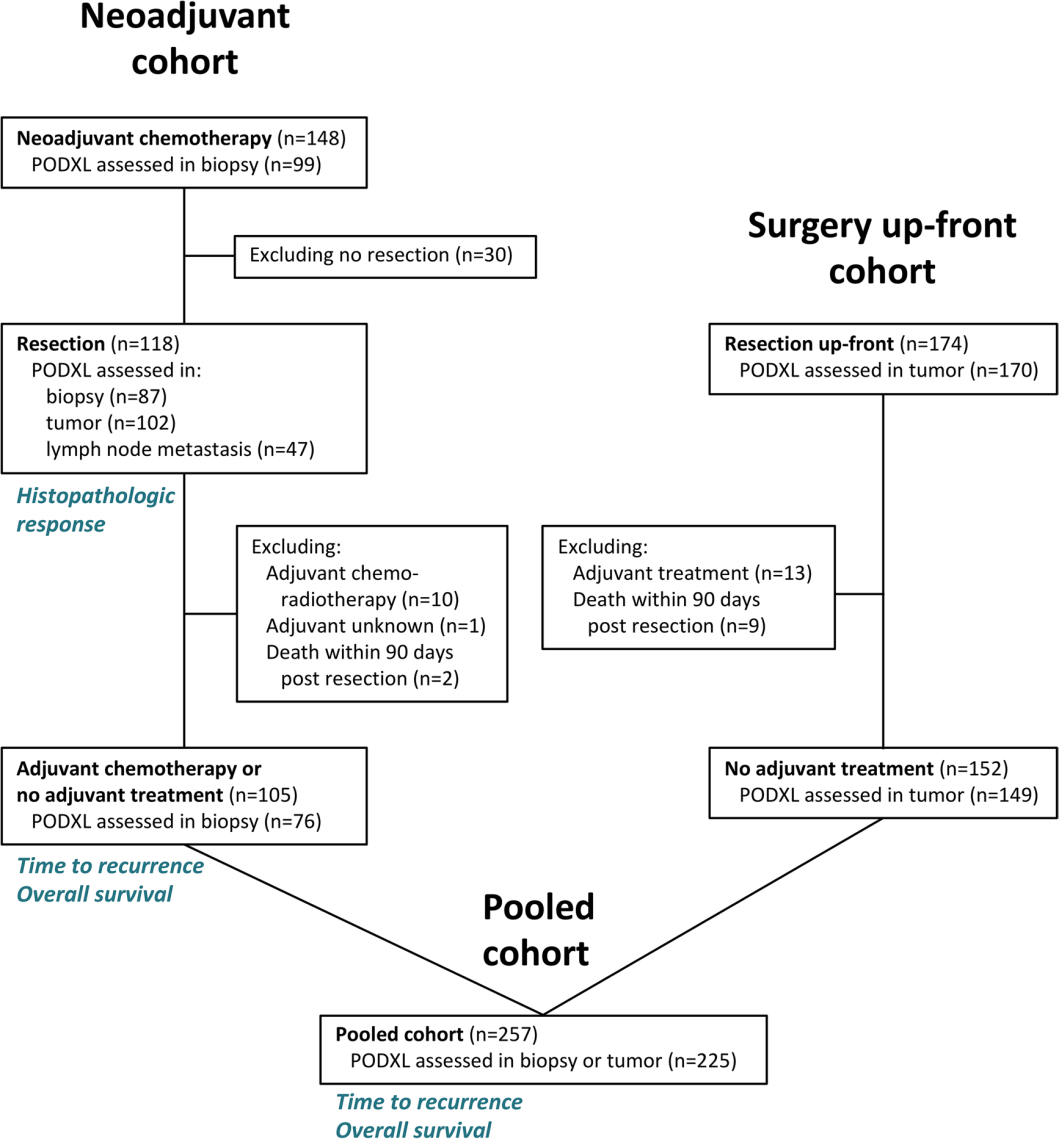
Overview of the cohorts depicting the number of patients and PODXL assessments. Biopsy refers to the pre-neoadjuvant biopsy from the primary tumor. Tumor and lymph node metastasis refers to TMA samples from the resected specimen

The surgery up-front cohort was derived from a previously described [29, 31–38] consecutive cohort of 174 patients treated with surgical resection between 2006 and 2010, without neoadjuvant therapy, but 13 patients who received adjuvant treatment were excluded. Follow-up was done until March 1, 2016. The neoadjuvant cohort and the surgery up-front cohort were merged into a pooled cohort. An overview of the cohorts is depicted in Fig. 1.

For both the neoadjuvant cohort and the surgery up-front cohort, clinical and pathological classification of tumor stage was done according to the 7th edition of the UICC/AJCC TNM classification, whereby tumors in the gastroesophageal junction Siewert type I–III were classified as esophageal cancer. Residual tumor status was classified as: R0 = no residual tumor (free resection margins according to the pathology report), R1 = possible microscopic residual tumor (narrow or compromised resection margins according to the pathology report), R2 = macroscopic residual tumor (according to the operative report).

### Tissue samples, immunohistochemistry and staining evaluation

From the neoadjuvant cohort, archival pre-neoadjuvant (diagnostic) biopsies were retrieved along with blocks from the post-neoadjuvant resected specimens. Tissue microarrays (TMAs) were constructed with duplicate cores from separate donor blocks from the resected primary tumor and (if available) from lymph node metastases as previously described [30]. For immunohistochemistry (IHC), 3 μm sections from the biopsies and 4 μm sections from the TMAs were prepared and stained using the rabbit polyclonal anti-PODXL antibody HPA002110 (Atlas Antibodies AB, Stockholm, Sweden) similarly as described in our previous study [29] on PODXL in the surgery up-front cohort. Staining of PODXL was scored as: negative (0), weak cytoplasmic positivity in any proportion of cells (1), moderate cytoplasmic positivity in any proportion of cells (2), distinct membranous positivity in ≤ 50% of cells (3) and distinct membranous positivity in > 50% of cells (4). For duplicate cores the highest score was used. Tumor-associated vascular endothelial cells and glomeruli in control renal tissue in the TMAs served as positive controls, and normal gastric mucosa and esophageal epithelium served as negative controls. Evaluation of the staining was carried out by two observers (AL and KJ) blinded to clinical and outcome data, and scoring discrepancies were discussed to reach consensus. Of note, for patients treated with surgery up-front, PODXL expression was assessed only in the resected primary tumor and not in lymph node metastasis which was done in our original report [29]. This resulted in an elevated proportion (from 17.0 to 21.2%) of cases denoted as PODXL negative in the surgery up-front cohort.

To validate the use of TMAs for PODXL scoring, whole tissue sections from 25 resected primary tumors were evaluated, blindly to the annotated scores of their corresponding TMA cores.

### Histopathologic response

The extent of residual cancer cells in the post-neoadjuvant resected primary tumor site was histologically evaluated using the 4-tiered tumor regression grading system described by Chirieac [39], i.e. 0%, 1–10%, 11–50% or > 50% residual cancer cells.

### Statistical analysis

Follow-up time was based on reverse Kaplan–Meier estimate. The scoring of PODXL expression was trichotomized into negative (0), low (1–2) or high (3–4) and then further dichotomized into negative (0) or positive (1–4). For assessment of the correlation of PODXL expression between tissue samples, Kendall’s tau-b (*τ*_b_) was used. Associations of clinicopathological factors with PODXL expression were analyzed using Kruskal–Wallis or Mann–Whitney *U* test for continuous variables, and Chi square test (Fisher’s exact for 2 × 2 tables and linear-by-linear association for larger tables) for categorical variables. Differences in histopathologic response stratified by trichotomized PODXL expression was assessed using Chi square test (linear-by-linear). Since only resected patients were included in the surgery up-front cohort, we assessed TTR and OS only in those patients in the neoadjuvant cohort who had undergone surgical resection. To account for the fact that a recurrence, by definition, cannot occur until the cancer has been resected, and for the differences in timing of surgery between the neoadjuvant cohort and the surgery up-front cohort, we used the resection date as baseline for TTR and OS. TTR was thus defined as time from resection to the date of biopsy or radiology proven recurrent disease. OS was defined as time from resection to the date of death. Furthermore, patients who died within 90 days from resection were excluded since this was regarded as being related to postoperative complications. Differences in Kaplan–Meier curves were assessed using log-rank test. Hazard ratios (HR) for TTR and OS were derived from Cox proportional-hazards regression. The variables adjusted for in the multivariable analyses are described in each corresponding table. In case of a low number of events per variable, only established prognostic variables were included in the adjusted model, in order to avoid overfitting of the data. In the Cox regression analyses of the pooled cohort, the potential role of PODXL as a predictive biomarker was assessed using an interaction term between the binary variables PODXL expression (negative vs. positive) × treatment (surgery only vs. neoadjuvant chemotherapy). A p-value < 0.05 was regarded as statistically significant and all tests were 2-sided. IBM^®^ SPSS^®^ Statistics version 23.0.0.2 for Mac OS was used for the statistical analyses.

## Results

### PODXL expression in tissue samples in the neoadjuvant cohort

**Fig. 2:**
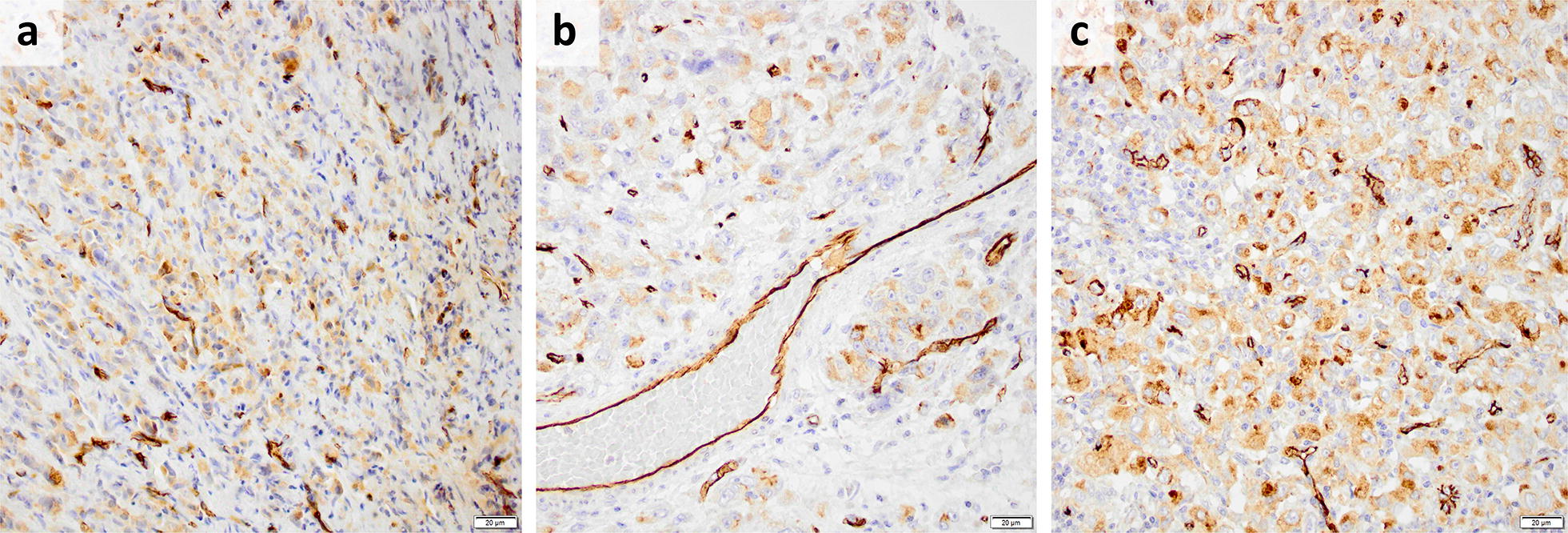
Sample IHC images of PODXL expression from a patient with adenocarcinoma in the gastroesophageal junction: **a** pre-neoadjuvant biopsy with moderate cytoplasmic positivity in tumor cells (score 2), **b** TMA core from post-neoadjuvant resected primary tumor with moderate cytoplasmic positivity in tumor cells (score 2), and **c** TMA core from post-neoadjuvant resected lymph node metastasis with distinct membranous positivity in ≤ 50% of tumor cells (score 3). Scale bar = 20 µm

PODXL expression could be assessed in pre-neoadjuvant biopsies from 99/148 (67%) patients, in post-neoadjuvant resected primary tumors from 102/118 (86%) patients, and in post-neoadjuvant resected lymph node metastases from 47/66 (71%) patients. Sample IHC images are shown in Fig. 2. The distribution of PODXL expression in the pre-neoadjuvant biopsies was: negative 33.3%, low 53.6% and high 13.1%. There was no correlation between PODXL expression in paired pre-neoadjuvant biopsies and resected primary tumors. There was a moderately strong correlation of PODXL expression between paired post-neoadjuvant resected primary tumors and lymph node metastases (trichotomized: *τ*_b_ = 0.48, p < 0.001, dichotomized: *τ*_b_ = 0.56, p < 0.001). The correlations and conversions of PODXL expression are further detailed in Additional file 2.

### Correlation of PODXL scoring between whole tissue sections and TMA cores from resected primary tumors

As shown in Additional file 3, there was a strong correlation between PODXL scoring in whole tissue sections and corresponding TMA cores (*τ*_b_ > 0.91, p < 0.001). In one of the 10 tested cases assessed as negative in the TMA, weak positive expression (score 1) was denoted on the whole tissue section.

### Associations of PODXL expression with clinicopathological factors in the neoadjuvant cohort

The only factor significantly associated with PODXL expression was post-neoadjuvant pathological T-stage (ypT), which was lower for patients with high PODXL expression in their pre-neoadjuvant biopsies (p = 0.029). For details see Additional file 4.

### Relationship between PODXL expression and histopathologic response to neoadjuvant chemotherapy

**Fig. 3:**
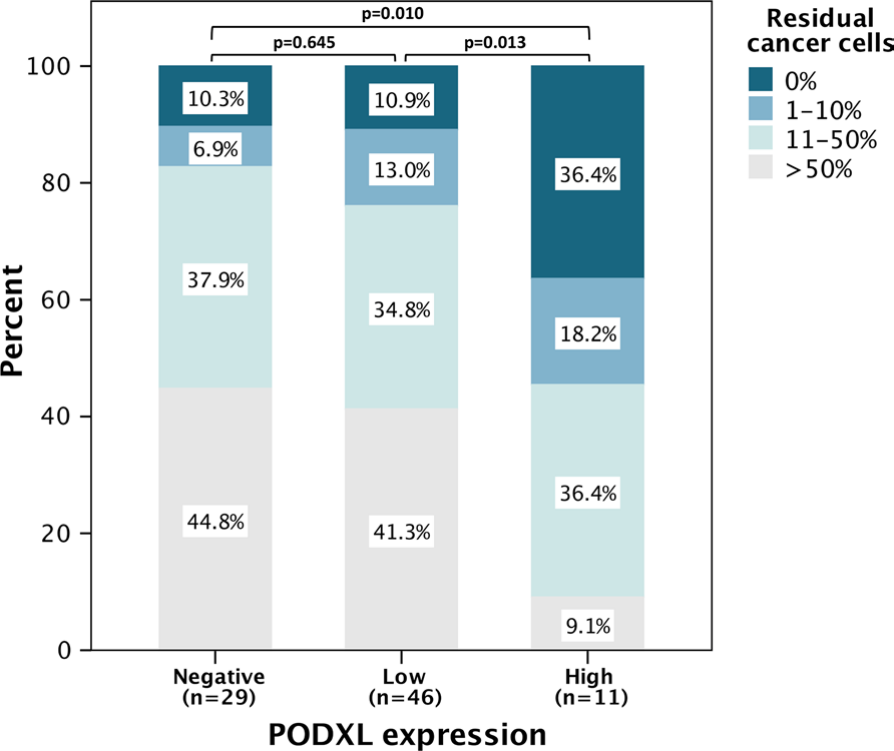
Distribution of histopathologic response (residual cancer cells) in the resected primary tumors across PODXL expression in the pre-neoadjuvant biopsies

As shown in Fig. 3, patients with high PODXL expression in their pre-neoadjuvant biopsies had a superior histopathologic response (notably 36% with no residual cancer cells) compared to those with negative (p = 0.010) or low (p = 0.013) PODXL expression. However, there were no significant differences in histopathological response between tumors with negative and low PODXL expression.

### Prognostic significance of PODXL expression in the neoadjuvant cohort

**Fig. 4:**
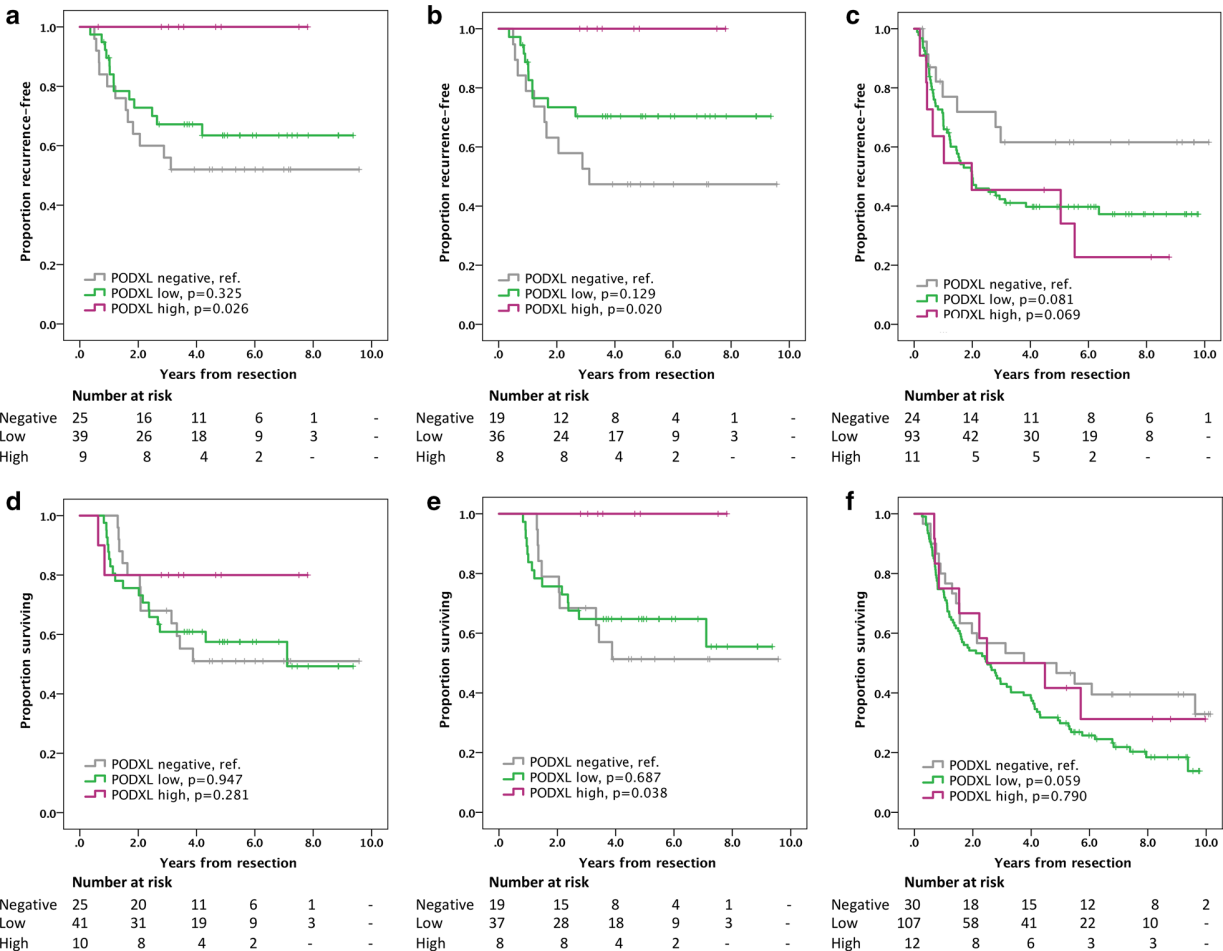
Kaplan-Meier plots of TTR (upper row) and OS (lower row) according to PODXL expression in patients treated with: (**a**, **d**) neoadjuvant ± adjuvant chemotherapy NOS, (**b**, **e**) neoadjuvant fluoropyrimidine and oxaliplatin ≥ 8 weeks ± adjuvant chemotherapy NOS, no irinotecan, or (**c**, **f**) surgery only

Kaplan–Meier plots and log-rank analyses (Fig. 4) demonstrate that patients with high PODXL expression in their pre-neoadjuvant biopsies were all recurrence-free at last follow-up, and had a superior TTR compared to PODXL negative cases (p = 0.026). Of note, one of the patients with a PODXL high tumor had an R2-resection, thus, TTR was not applicable in this case. Furthermore, for patients with PODXL high tumors treated with neoadjuvant fluoropyrimidine and oxaliplatin for a minimum of 8 weeks, no deaths had occurred at last follow-up, and OS was superior compared to PODXL negative cases (p = 0.038). There were no statistical differences between PODXL negative and PODXL low cases for neither TTR nor OS. As a comparison, survival plots stratified by PODXL expression in resected primary tumors from the surgery up-front cohort are also shown in Fig. 4.

Cox regression analyses on the neoadjuvant cohort for TTR and OS could not be performed with trichotomized PODXL expression due to few events (mostly none) for PODXL high tumors, thus dichotomized PODXL expression was used (see tables in Additional file 5). PODXL was not prognostic neither for TTR nor OS, except in patients treated with neoadjuvant fluoropyrimidine and oxaliplatin for a minimum of 8 weeks, where PODXL was prognostic for TTR in univariable analysis (HR 0.40, 95% CI 0.17–0.96, p = 0.041), but not when adjusting for known prognostic factors.

### Associations of PODXL expression with clinicopathological factors in the pooled cohort

The pooled cohort, stratified by PODXL expression, was well balanced for all clinicopathological factors (Table 2), except for a larger proportion of gastric cancer in PODXL negative compared to positive cases (58.2 vs. 40.0%, p = 0.020), and a larger proportion treated with neoadjuvant chemotherapy in PODXL negative compared to positive cases (45.5 vs. 30.0%, p = 0.048).

**Table 2:** Associations of PODXL expression with clinicopathological factors in the pooled cohort

	PODXL negative n (%)	PODXL positive n (%)	p
N	55	170	
Age (years)			
Mean	69.5	67.3	0.250
Median	68.5	67.7	
Range	42.6–88.8	21.1–94.4	
Sex			
Female	20 (36.4)	43 (25.3)	0.122
Male	35 (63.6)	127 (74.7)	
Location			
Esophagus	23 (41.8)	102 (60.0)	*0.020*
Stomach	32 (58.2)	68 (40.0)	
cT stage			
T1	1 (1.8)	7 (4.1)	0.413
T2	31 (56.4)	75 (44.1)	
T3	22 (40.0)	85 (50.0)	
T4	1 (1.8)	3 (1.8)	
cN stage			
N0	39 (70.9)	110 (64.7)	0.422
N1	12 (21.8)	43 (25.3)	
N2	3 (5.5)	14 (8.2)	
N3	1 (1.8)	3 (1.8)	
cM stage			
M0	51 (92.7)	161 (94.7)	0.525
M1	4 (7.3)	9 (5.3)	
Differentiation			
Low grade	3 (5.5)	6 (3.6)	0.241
Intermediate grade	23 (41.8)	59 (35.1)	
High grade	29 (52.7)	103 (61.3)	
Missing data		2	
Lauren classification			
Intestinal	42 (76.4)	109 (64.5)	0.274
Mixed	0	15 (8.9)	
Diffuse	13 (23.6)	45 (26.6)	
Missing data		1	
Residual tumor status			
R0	45 (81.8)	128 (75.3)	0.392
R1	8 (14.5)	35 (20.6)	
R2	2 (3.6)	7 (4.1)	
Number of examined nodes			
Mean	30	34	0.149
Median	28	32	
Range	5–85	1–112	
Treatment			
Surgery only	30 (54.5)	119 (70.0)	*0.048*
Neoadjuvant chemotherapy^a^	25 (45.5)	51 (30.0)	
Follow-up (years from resection)			
Median (95% CI)	7.2 (6.6–7.7)	7.3 (6.7–7.6)	0.125

^a^Neoadjuvant chemotherapy NOS ± adjuvant chemotherapy NOS

### Predictive role of PODXL in the pooled cohort

**Fig. 5:**
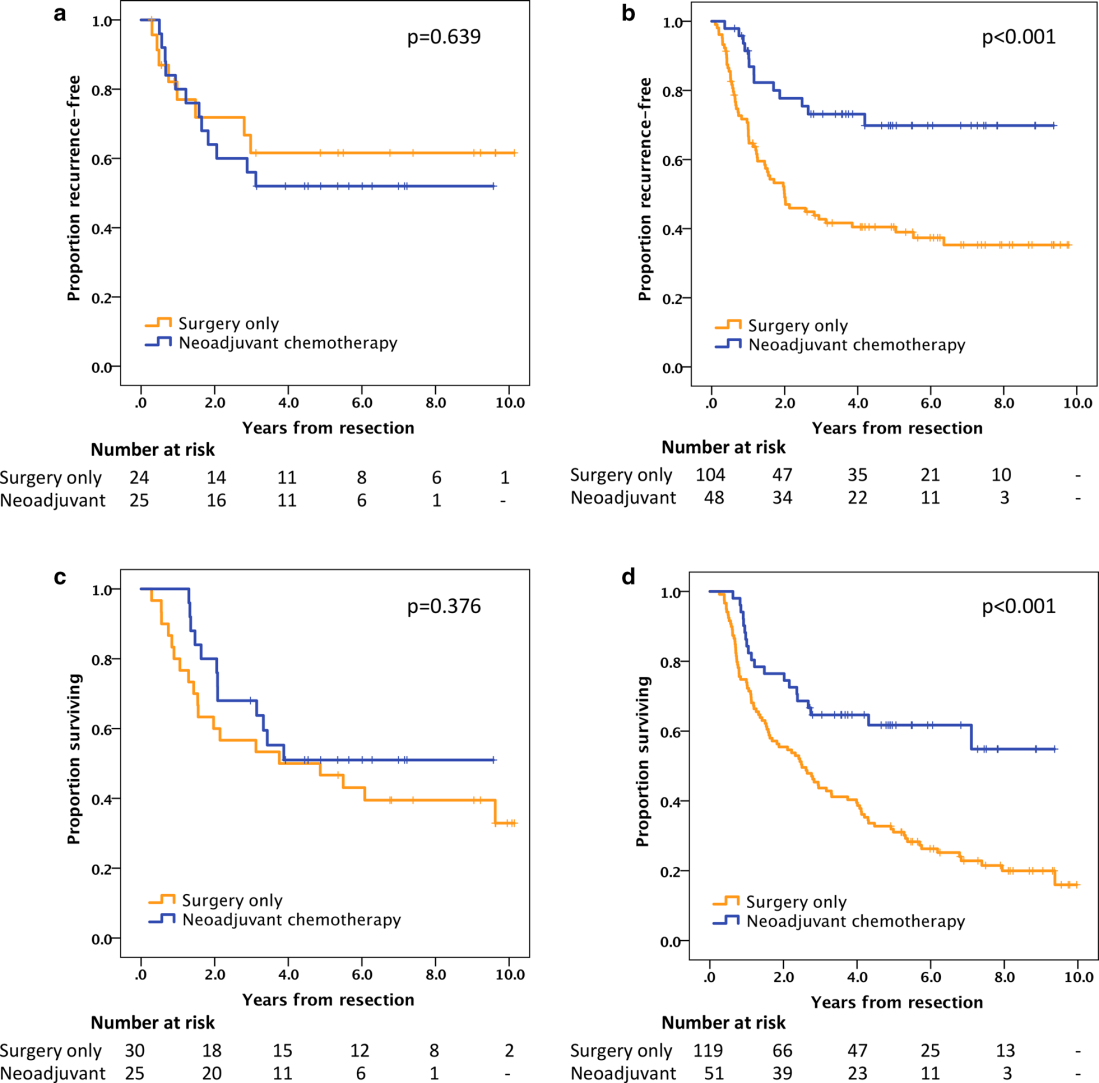
Kaplan-Meier plots of TTR (upper row) and OS (lower row) according to treatment (surgery only vs. neoadjuvant chemotherapy NOS ± adjuvant chemotherapy NOS) in patients with: (**a**, **c**) PODXL negative tumors, or (**b**, **d**) PODXL positive tumors

Kaplan–Meier analyses (Fig. 5) demonstrate that for patients with PODXL negative tumors in the pre-neoadjuvant biopsies, neoadjuvant ± adjuvant chemotherapy had no effect on TTR or OS compared to surgery alone. In contrast, for PODXL positive cases, a superior TTR (estimated recurrence-free rate at 5 years: 69% vs. 41%) as well as OS (estimated surviving rate at 5 years: 61% vs. 31%) was shown with neoadjuvant ± adjuvant chemotherapy compared to surgery alone. Cox regression analyses of TTR and OS (Table 3) stratified by PODXL expression were consistent with these findings in both univariable and multivariable analyses. Furthermore, in Cox regression analyses of the entire (unstratified) pooled cohort, a beneficial effect of neoadjuvant ± adjuvant chemotherapy on TTR and OS was seen only in univariable analyses but not when adjusting for other factors including an interaction term (PODXL expression x treatment), thus indicating a possible predictive role for PODXL. The interaction term was statistically significant for TTR in unadjusted analysis for neoadjuvant chemotherapy NOS (not otherwise specified) ± adjuvant chemotherapy NOS vs. surgery alone (p = 0.018) but not in the adjusted analysis (p = 0.069). Limiting the neoadjuvant chemotherapy group to those treated with neoadjuvant fluoropyrimidine and oxaliplatin for a minimum of 8 weeks, the interaction term was significant in both unadjusted (p = 0.006) and adjusted (p = 0.024) analyses, further supporting a potential predictive role for PODXL. For OS, however, the interaction term was not significant, neither in unadjusted nor in adjusted analyses.

**Table 3:** Hazard ratios for recurrence (TTR) and death (OS) in the pooled cohort

	TTR					OS				
	n (events)	Unadjusted		Adjusted^c^		n (events)	Unadjusted		Adjusted^d^	
		HR (95% CI)	p	HR (95% CI)	p		HR (95% CI)	p	HR (95% CI)	p
PODXL negative										
Treatment										
Surgery only	24 (8)					30 (19)				
Neoadjuvant chemotherapy^a^	25 (12)	1.24 (0.51–3.03)	0.639	0.85 (0.31–2.39)	0.763	25 (12)	0.72 (0.35–1.50)	0.378	0.88 (0.31–2.49)	0.807
Neoadjuvant fp + oxa ≥ 8 w^b^	19 (10)	1.39 (0.55–3.52)	0.490	0.79 (0.26–2.34)	0.665	19 (9)	0.74 (0.33–1.64)	0.453	0.77 (0.25–2.34)	0.638

	TTR					OS				
	n (events)	Unadjusted		Adjusted^c^		n (events)	Unadjusted		Adjusted^d^	
		HR (95% CI)	p	HR (95% CI)	p		HR (95% CI)	p	HR (95% CI)	p
PODXL positive										
Treatment										
Surgery only	104 (62)					119 (93)				
Neoadjuvant chemotherapy^a^	48 (13)	0.35 (0.19–0.64)	*0.001*	0.31 (0.16–0.59)	*< 0.001*	51 (20)	0.43 (0.26–0.69)	*0.001*	0.65 (0.38–1.10)	0.110
Neoadjuvant fp + oxa ≥ 8 w^b^	44 (10)	0.29 (0.15–0.57)	*< 0.001*	0.27 (0.13–0.55)	*< 0.001*	45 (14)	0.32 (0.18–0–56)	*< 0.001*	0.48 (0.26–0.90)	*0.021*

	TTR					OS				
	n (events)	Unadjusted		Adjusted^e^		n (events)	Unadjusted		Adjusted^e^	
		HR (95% CI)	p	HR (95% CI)	p		HR (95% CI)	p	HR (95% CI)	p
Entire pooled cohort										
Treatment										
Surgery only	131 (72)					152 (114)				
Neoadjuvant chemotherapy^a^	102 (41)	0.64 (0.43–0.93)	*0.021* *0.018* ^†^	1.01 (0.40–2.55)	0.986 0.069†	105 (49)	0.57 (0.41–0.80)	*< 0.001* 0.191^†^	0.85 (0.40–1.81)	0.669 0.640^†^
Neoadjuvant fp + oxa ≥ 8 w^b^	87 (33)	0.60 (0.40–0.91)	*0.016* *0.006* ^†^	1.18 (0.45–3.10)	*0.733 0.024* ^†^	88 (36)	0.50 (0.34–0.72)	*< 0.001* 0.080^†^	0.84 (0.37–1.94)	0.690 0.395^†^

^†^p for interaction term (PODXL expression × treatment)

^a^Neoadjuvant chemotherapy NOS ± adjuvant chemotherapy NOS

^b^Neoadjuvant fluoropyrimidine + oxaliplatin ≥ 8 weeks ± adjuvant chemotherapy NOS, no irinotecan

^c^Adjusted for cT, cN, residual tumor status and treatment

^d^Adjusted for age, cT, cN, residual tumor status and treatment

^e^Adjusted for age, sex, location, cT, cN, cM, differentiation grade, Lauren classification, residual tumor status, PODXL status, treatment and interaction term

## Discussion

The present study demonstrates a superior prognosis for patients with gastric or esophageal adenocarcinoma with high PODXL expression, treated with neoadjuvant ± adjuvant fluoropyrimidine- and oxaliplatin-based chemotherapy, compared to those with negative or low PODXL expression. This is in contrast to our previous findings [29] of PODXL being an independent prognostic biomarker for poor survival in gastric and esophageal adenocarcinoma treated with surgery up-front, thus suggesting a possible predictive role for PODXL. This hypothesis was further tested in analyses of a pooled cohort, whereby it was demonstrated that patients with PODXL negative tumors had no obvious benefit of chemotherapy, in contrast to patients with PODXL positive tumors. The potential predictive role of PODXL was further strengthened for TTR, but not for OS, in the interaction analyses. The above findings of PODXL as a potential predictive biomarker for benefit of neoadjuvant ± adjuvant chemotherapy is in line with results from previous studies on colorectal [14] and periampullary [17] cancer, showing that only patients with PODXL high tumors seem to benefit from adjuvant chemotherapy.

In addition, an association between PODXL expression and histopathologic response to neoadjuvant chemotherapy was found, further supporting the potential role of PODXL as a predictive biomarker. However, the mechanistic basis underlying these observations warrants further studies, not least since in vitro data on cell lines from colon cancer [26], osteosarcoma [40], oral tongue squamous cell carcinoma [41] and astrocytoma [42], have demonstrated links between PODXL and resistance to chemotherapy. It would also be of interest to assess whether there is a relationship between PODXL expression and the molecular subtypes proposed by The Cancer Genome Atlas for gastric [43] and esophageal [44] adenocarcinoma.

In the neoadjuvant cohort, merely 80% of the patients underwent resection which is probably attributable to initial understaging since diagnostic laparoscopy was not part of routine work-up, and thus, non-resectable disease was not found until surgery was commenced. Although the intention was to treat all patients with neoadjuvant chemotherapy + adjuvant treatment, only 66.7% of the resected patients started adjuvant chemotherapy and 8.5% started adjuvant chemoradiotherapy. This is in line with the pivotal randomized trials on perioperative chemotherapy where only 49.5% started adjuvant chemotherapy in the FFCD 9703 trial [6] and 65.6% in the MAGIC trial [5]. Since the value of the adjuvant part of perioperative chemotherapy is unclear, and results from retrospective studies [45, 46] are conflicting, we focused on the neoadjuvant part.

For a predictive biomarker to be useful in the neoadjuvant setting, it must be available prior to initiation of treatment, thus analysis of the diagnostic biopsies is key. In our study, the quality of the diagnostic biopsies was rather poor, and as shown, not all of them were suitable for assessment of PODXL expression. Therefore, improved biopsy sampling is advocated in future studies. In addition, if negative PODXL expression is to be used to select patients for surgery alone, it would be of importance to account for possible intratumor heterogeneity of PODXL expression, and thus take multiple biopsies from different parts of the tumor. Of note, since PODXL is also expressed in tumor-associated vasculature, immunohistochemistry is the preferable assay for analysis of PODXL expression in a clinically relevant context. In a previous study on colorectal cancer, there was no significant correlation between mRNA levels and protein expression of PODXL, and only the latter carried prognostic information [15].

A limitation to this study is its retrospective nature and, ideally, the predictive value of PODXL should be assessed in a randomized trial. However, since the addition of oncological treatment to surgery is the current standard worldwide, a prospective trial with surgery alone as control arm is not feasible at present. Another weakness is that the assessment of PODXL expression in the pooled cohort was based on pre-neoadjuvant biopsies in the neoadjuvant group, and on TMA samples from the resected tumor in the surgery only group. Whether this discrepancy in sampling procedures may have affected our results is unclear. Unfortunately, we did not have access to the diagnostic biopsies for the patients treated with surgery up-front.

## Conclusions

In summary, patients with resectable gastric or esophageal adenocarcinoma with high PODXL expression in their diagnostic biopsies have an excellent prognosis when treated with neoadjuvant ± adjuvant fluoropyrimidine- and oxaliplatin-based chemotherapy. Furthermore, and importantly, it is suggested that PODXL expression is predictive for benefit of chemotherapy in this setting. If the results from this study can be confirmed in subsequent studies, it would have a substantial impact on daily clinical practise in that patients with PODXL negative tumors could be spared chemotherapy and treated with surgery alone.

## Additional files

**Additional file 1: Table S1.** Description of chemotherapy regimens in the neoadjuvant cohort.

**Additional file 2: Table S2.** Correlation and conversion of PODXL expression between paired tissue samples in the neoadjuvant cohort.

**Additional file 3: Table S3.** Correlation of PODXL score/expression between whole tissue sections and corresponding TMA cores from resected primary tumors.

**Additional file 4: Table S4.** Associations of PODXL expression with clinicopathological factors in the neoadjuvant cohort.

**Additional file 5: Table S5.** Cox regression for TTR and OS in the neoadjuvant cohort.

## Abbreviations

CI: confidence interval
HR: hazard ratio
IHC: immunohistochemistry
NOS: not otherwise specified
OS: overall survival
PODXL: podocalyxin-like protein 1
TMA: tissue microarray
TTR: time to recurrence
