# Supplementary material for: Podocalyxin-like protein as a predictive biomarker for benefit of neoadjuvant chemotherapy in resectable gastric and esophageal adenocarcinoma
Source: J Transl Med. 2018 Oct 24;16:290. doi: 10.1186/s12967-018-1668-3 (PMC6201481; doi:10.1186/s12967-018-1668-3)
Supplement: Supplementary file 1 — Additional file 1: Table S1. Description of chemotherapy regimens in the neoadjuvant cohort. [file 12967_2018_1668_MOESM1_ESM.docx]

| **Additional file 1: Table S1a**  **Chemotherapy regimens in the neoadjuvant cohort** | | |
| --- | --- | --- |
|  | **Neoadjuvant (n=148)**  n (%) | **Adjuvant**  **(n=78)**  n (%) |
| ECX  EOX  EOX → ECX  EOX → FOLFOX  EOX → FLOX  FOLFOX  FOLFOX → FLOX  FLOX  FLOX → FOLFOX  FOLFIRI  FLIRI  XELIRI  EOX → FLIRI  FLOX → FLIRI  5-FU de Gramont  FLV | 1 (0.7)  83 (56.1)  1 (0.7)  12 (8.1)  1 (0.7)  31 (20.9)  1 (0.7)  10 (6.8)  1 (0.7)  1 (0.7)  2 (1.4)  1 (0.7)  2 (1.4)  1 (0.7) | 1 (1.3)  30 (38.5)  2 (2.6)  2 (2.6)  23 (29.5)  1 (1.3)  12 (15.4)  1 (1.3)  3 (3.8)  2 (2.6)  1 (1.3) |
| Treatment duration (weeks)  Mean  Median  Range | 8.3  9.0  1-14 | 7.5  8.0  2-11 |
| ECX: epirubicin 50 mg/m^2^ day 1, cisplatin 60 mg/m^2^ day 1, capecitabine 1000 mg/m^2^ x 2 day 1-14, cycle length 21 days  EOX: epirubicin 50 mg/m^2^ day 1, oxaliplatin 130 mg/m^2^ day 1, capecitabine 625 mg/m^2^ x 2 day 1-21, cycle length 21 days  FOLFOX: fluorouracil 2400 mg/m^2^ 44 h iv infusion, fluorouracil 400 mg/m^2^ iv push day 1, calcium folinate 200 mg/m^2^ day 1, oxaliplatin 85 mg/m^2^ day 1, cycle length 14 days  FLOX: fluorouracil 500 mg/m^2^ iv push day 1 and 2, calcium folinate 60 mg/m^2^ day 1 and 2, oxaliplatin 85 mg/m^2^ day 1, cycle length 14 days  FOLFIRI: fluorouracil 2400 mg/m^2^ 44 h iv infusion, fluorouracil 400 mg/m^2^ iv push day 1, calcium folinate 200 mg/m^2^ day 1, irinotecan 180 mg/m^2^ day 1, cycle length 14 days  FLIRI: fluorouracil 500 mg/m^2^ iv push day 1 and 2, calcium folinate 60 mg/m^2^ day 1 and 2, irinotecan 180 mg/m^2^ day 1, cycle length 14 days  XELIRI: capecitabine 1000 mg/m^2^ x 2 day 1-14, irinotecan 180 mg/m^2^ day 1, cycle length 21 days  5-FU de Gramont: fluorouracil 2400 mg/m^2^ 44 h iv infusion, fluorouracil 400 mg/m^2^ iv push day 1, calcium folinate 200 mg/m^2^ day 1, cycle length 14 days  FLV: fluorouracil 500 mg/m^2^ iv push day 1 and 2, calcium folinate 60 mg/m^2^ day 1 and 2, cycle length 14 days | | |

| **Additional file 1: Table S1b**  **Chemotherapy regimens in resected cases not dead within 90 days post surgery, PODXL not missing in biopsy** | | |
| --- | --- | --- |
|  | **Neoadjuvant (n=76)**  n (%) | **Adjuvant (n=54)**  n (%) |
| EOX  EOX → FOLFOX  EOX → FLOX  FOLFOX  FOLFOX → FLOX  FLOX  FLOX → FOLFOX  FLIRI  EOX → FLIRI  5-FU de Gramont | 47 (61.8)  9 (11.8)  1 (1.3)  10 (13.2)  6 (7.9)  1 (1.3)  1 (1.3)  1 (1.3) | 23 (42.6)  2 (3.7)  1 (1.9)  15 (27.8)  1 (1.9)  8 (14.8)  2 (3.7)  2 (3.7) |
| Treatment duration (weeks)  Mean  Median  Range | 8.6  9.0  3-14 | 7.5  8.0  2-11 |
