# Supplementary material for: Podocalyxin-like protein as a predictive biomarker for benefit of neoadjuvant chemotherapy in resectable gastric and esophageal adenocarcinoma
Source: J Transl Med. 2018 Oct 24;16:290. doi: 10.1186/s12967-018-1668-3 (PMC6201481; doi:10.1186/s12967-018-1668-3)
Supplement: Supplementary file 2 — Additional file 2: Table S2. Correlation and conversion of PODXL expression between paired tissue samples in the neoadjuvant cohort. [file 12967_2018_1668_MOESM2_ESM.docx]

| **Additional file 2: Table S2a**  **Correlation and conversion of PODXL expression (trichotomized) between paired tissue samples in the neoadjuvant cohort** | | | | | | | | | |
| --- | --- | --- | --- | --- | --- | --- | --- | --- | --- |
|  | **PODXL in pre-neoadjuvant biopsy** | | | |  | **PODXL in post-neoadjuvant resected**  **lymph node metastasis** | | | |
| **PODXL in post-neoadjuvant resected primary tumor** | Negative | Low | High | Correlation (*τ*_b)_  p  Conversion (any) | **PODXL in post-neoadjuvant resected primary tumor** | Negative | Low | High | Correlation (*τ*_b)_  p  Conversion (any) |
| Negative | 8  10.8% | 17  23.0% | 1  1.4% | 0.003  0.981  58.2% | Negative | 12  28.6% | 3  7.1% | 0 | 0.484  **<0.001**  30.9% |
| Low | 16  21.6% | 22  29.7% | 6  8.1% |  | Low | 4  9.5% | 15  35.7% | 3  7.1% |  |
| High | 2  2.7% | 1  1.4% | 1  1.4% |  | High | 2  4.8% | 1  2.4% | 2  4.8% |  |

| **Additional file 2: Table S2b**  **Correlation and conversion of PODXL expression (dichotomized) between paired tissue samples in the neoadjuvant cohort** | | | | | | | |
| --- | --- | --- | --- | --- | --- | --- | --- |
|  | **PODXL in pre-neoadjuvant biopsy** | | |  | **PODXL in post-neoadjuvant resected**  **lymph node metastasis** | | |
| **PODXL in post-neoadjuvant resected primary tumor** | Negative | Positive | Correlation (*τ*_b)_  p  Conversion (any) | **PODXL in post-neoadjuvant resected primary tumor** | Negative | Positive | Correlation (*τ*_b)_  p  Conversion (any) |
| Negative | 8  10.8% | 18  24.3% | -0.067  0.556  48.6% | Negative | 12  28.6% | 3  7.1% | 0.559  **<0.001**  21.4% |
| Positive | 18  24.3% | 30  40.5% |  | Positive | 6  14.3% | 21  50.0% |  |
