# Supplementary material for: Podocalyxin-like protein as a predictive biomarker for benefit of neoadjuvant chemotherapy in resectable gastric and esophageal adenocarcinoma
Source: J Transl Med. 2018 Oct 24;16:290. doi: 10.1186/s12967-018-1668-3 (PMC6201481; doi:10.1186/s12967-018-1668-3)
Supplement: Supplementary file 3 — Additional file 3: Table S3. Correlation of PODXL score/expression between whole tissue sections and corresponding TMA cores from resected primary tumors. [file 12967_2018_1668_MOESM3_ESM.docx]

| **Addiitonal file 3: Table S3a**  **Correlation of PODXL score between whole tissue sections and corresponding TMA cores from resected primary tumors** | | | | | | |
| --- | --- | --- | --- | --- | --- | --- |
|  | **PODXL score in TMA cores** | | | | | |
| **PODXL score in whole tissue sections** | 0 | 1 | 2 | 3 | 4 | Correlation (*τ*_b)_  p |
| 0 | 9  36.0% | 0  0.0% | 0  0.0% | 0  0.0% | 0  0.0% | 0.944  **<0.001** |
| 1 | 1  4.0% | 4  16.0% | 0  0.0% | 0  0.0% | 0  0.0% |  |
| 2 | 0  0.0% | 1  4.0% | 4  16.0% | 0  0.0% | 0  0.0% |  |
| 3 | 0  0.0% | 0  0.0% | 0  0.0% | 4  16.0% | 1  4.0% |  |
| 4 | 0  0.0% | 0  0.0% | 0  0.0% | 0  0.0% | 1  4.0% |  |

| **Addiitonal file 3: Table S3b**  **Correlation of PODXL expression (trichotomized/dichotomized) between whole tissue sections and corresponding TMA cores from resected primary tumors** | | | | | | | | |
| --- | --- | --- | --- | --- | --- | --- | --- | --- |
|  | **PODXL (trichotomized) in TMA cores** | | | |  | **PODXL (dichotomized) in TMA cores** | | |
| **PODXL (trichotomized) in whole tissue sections** | Negative | Low | High | Correlation (*τ*_b)_  p | **PODXL (dichotomized) in whole tissue sections** | Negative | Positive | Correlation (*τ*_b)_  p |
| Negative | 9  36.0% | 0  0.0% | 0  0.0% | 0.956  **<0.001** | Negative | 9  36.0% | 0  0.0% | 0.919  **<0.001** |
| Low | 1  4.0% | 9  36.0% | 0  0.0% |  |  |  |  |  |
|  |  |  |  |  | Positive | 1  4.0% | 15  60.0% |  |
| High | 0  0.0% | 0  0.0% | 6  24.0% |  |  |  |  |  |
