# Supplementary material for: Podocalyxin-like protein as a predictive biomarker for benefit of neoadjuvant chemotherapy in resectable gastric and esophageal adenocarcinoma
Source: J Transl Med. 2018 Oct 24;16:290. doi: 10.1186/s12967-018-1668-3 (PMC6201481; doi:10.1186/s12967-018-1668-3)
Supplement: Supplementary file 4 — Additional file 4: Table S4. Associations of PODXL expression with clinicopathological factors in the neoadjuvant cohort. [file 12967_2018_1668_MOESM4_ESM.docx]

| **Additional file 4: Table S4a**  **Associations of PODXL expression (trichotomized) with clinicopathological factors in the neoadjuvant cohort, resected patients not dead within 90 days post surgery** | | | | |
| --- | --- | --- | --- | --- |
|  | **PODXL negative**  n (%) | **PODXL low**  n (%) | **PODXL high**  n (%) | p |
| **N** | 25 (33) | 41 (54) | 10 (13) |  |
| **Age**  mean  median  range | 65.1  65.0  46.5-81.0 | 62.1  65.8  21.1-78.5 | 60.8  59.0  47.6-75.4 | 0.443 |
| **Sex**  Female  Male | 13 (52.0)  12 (48.0) | 14 (34.1)  27 (65.9) | 4 (40.0)  6 (60.0) | 0.303 |
| **Location**  Esophagus  Stomach | 9 (36.0)  16 (64.0) | 18 (43.9)  23 (56.1) | 4 (40.0)  6 (60.0) | 0.690 |
| **cT stage**  T1  T2  T3  T4 | 1 (4.0)  12 (48.0)  11 (44.0)  1 (4.0) | 0  13 (31.7)  25 (61.0)  3 (7.3) | 0  6 (60.0)  4 (40.0) | 0.712 |
| **cN stage**  N0  N1  N2  N3 | 14 (56.0)  9 (36.0)  1 (4.0)  1 (4.0) | 23 (56.1)  11 (26.8)  4 (9.8)  3 (7.3) | 8 (80.0)  1 (10.0)  1 (10.0)  0 | 0.660 |
| **cM stage**  M0  M1 | 23 (92.0)  2 (8.0) | 36 (87.8)  5 (12.2) | 10 (100.0) | 0.707 |
| **Differentiation grade**  Low grade  Intermediate grade  High grade | 1 (4.0)  13 (52.0)  11 (44.0) | 2 (4.9)  17 (41.5)  22 (53.7) | 0  5 (62.5)  3 (37.5) | 0.852 |
| **Lauren classification**  Intestinal  Mixed  Diffuse | 18 (72.0)  0  7 (28.0) | 22 (53.7)  5 (12.2)  14 (34.1) | 5 (55.6)  1 (11.1)  3 (33.3) | 0.376 |
| **Neoadjuvant** **fp + oxa ≥8 w of both, no irinotecan**  No  Yes | 6 (24.0)  19 (76.0) | 4 (9.8)  37 (90.2) | 2 (20.0)  8 (80.0) | 0.432 |
| **Adjuvant chemotherapy**  No  Yes | 6 (24.0)  19 (76.0) | 11 (26.8)  30 (73.2) | 5 (50.0)  5 (50.0) | 0.196 |
| **R classification**  R0  R1  R2 | 20 (80.0)  5 (20.0)  0 | 36 (87.8)  4 (9.8)  1 (2.4) | 9 (90.0)  0  1 (10.0) | 0.863 |
| **ypT stage**  T0  T1  T2  T3  T4 | 3 (12.0)  5 (20.0)  2 (8.0)  8 (32.0)  7 (28.0) | 5 (12.5)  6 (15.0)  6 (15.0)  16 (40.0)  7 (17.5) | 4 (40.0)  1 (10.0)  5 (50.0)  0  0 | **0.029** |
| **ypN stage**  N0  N1  N2  N3 | 14 (56.0)  5 (20.0)  2 (8.0)  4 (16.0) | 18 (43.9)  12 (29.3)  8 (19.5)  3 (7.3) | 7 (70.0)  3 (30.0)  0  0 | 0.289 |
| **ypM stage**  M0  M1 | 23 (92.0)  2 (8.0) | 40 (97.6)  1 (2.4) | 10 (100.0)  0 | 0.204 |

| **Additional file 4: Table S4b**  **Associations of PODXL expression (dichotomized) with clinicopathological factors in the neoadjuvant cohort, resected patients not dead within 90 days post surgery** | | | |
| --- | --- | --- | --- |
|  | **PODXL negative**  n (%) | **PODXL positive**  n (%) | p |
| **N** | 25 (33) | 51 (67) |  |
| **Age**  mean  median  range | 65.1  65.0  46.5-81.0 | 61.8  63.7  21.1-78.5 | 0.362 |
| **Sex**  Female  Male | 13 (52.0)  12 (48.0) | 18 (35.3)  33 (64.7) | 0.216 |
| **Location**  Esophagus  Stomach | 9 (36.0)  16 (64.0) | 22 (43.1)  29 (56.9) | 0.625 |
| **cT stage**  T1  T2  T3  T4 | 1 (4.0)  12 (48.0)  11 (44.0)  1 (4.0) | 0  19 (37.9)  29 (56.9)  3 (5.9) | 0.166 |
| **cN stage**  N0  N1  N2  N3 | 14 (56.0)  9 (36.0)  1 (4.0)  1 (4.0) | 31 (60.8)  12 (23.5)  5 (9.8)  3 (5.9) | 0.818 |
| **cM stage**  M0  M1 | 23 (92.0)  2 (8.0) | 46 (90.2)  5 (9.8) | 1.000 |
| **Differentiation grade**  Low grade  Intermediate grade  High grade | 1 (4.0)  13 (52.0)  11 (44.0) | 2 (4.1)  22 (44.9)  25 (51.0) | 0.624 |
| **Lauren classification**  Intestinal  Mixed  Diffuse | 18 (72.0)  0  7 (28.0) | 27 (54.0)  6 (12.0)  17 (34.0) | 0.289 |
| **Neoadjuvant** **fp + oxa ≥8 w of both, no irinotecan**  No  Yes | 6 (24.0)  19 (76.0) | 6 (11.8)  45 (88.2) | 0.193 |
| **Adjuvant chemotherapy**  No  Yes | 6 (24.0)  19 (76.0) | 16 (31.4)  35 (68.6) | 0.597 |
| **R classification**  R0  R1  R2 | 20 (80.0)  5 (20.0)  0 | 45 (88.2)  4 (7.8)  2 (3.9) | 0.691 |
| **ypT stage**  T0  T1  T2  T3  T4 | 3 (12.0)  5 (20.0)  2 (8.0)  8 (32.0)  7 (28.0) | 9 (18.0)  7 (14.0)  11 (22.0)  16 (32.0)  7 (14.0) | 0.307 |
| **ypN stage**  N0  N1  N2  N3 | 14 (56.0)  5 (20.0)  2 (8.0)  4 (16.0) | 25 (49.0)  15 (29.4)  8 (15.7)  3 (5.9) | 0.818 |
| **ypM stage**  M0  M1 | 23 (92.0)  2 (8.0) | 50 (98.0)  1 (2.0) | 0.250 |
