# Supplementary material for: Podocalyxin-like protein as a predictive biomarker for benefit of neoadjuvant chemotherapy in resectable gastric and esophageal adenocarcinoma
Source: J Transl Med. 2018 Oct 24;16:290. doi: 10.1186/s12967-018-1668-3 (PMC6201481; doi:10.1186/s12967-018-1668-3)
Supplement: Supplementary file 5 — Additional file 5: Table S5. Cox regression for TTR and OS in the neoadjuvant cohort. [file 12967_2018_1668_MOESM5_ESM.docx]

| **Additional file 5: Table S5a**  **Cox regression**  **Neoadjuvant chemotherapy NOS ± adjuvant chemotherapy NOS** | | | | | | | | | | |
| --- | --- | --- | --- | --- | --- | --- | --- | --- | --- | --- |
|  | **TTR** | | | | | **OS** | | | | |
|  |  | **Unadjusted** | | **Adjusted^1^** | |  | **Unadjusted** | | **Adjusted^2^** | |
|  | n (events) | HR (95% CI) | p | HR (95% CI) | p | n (events) | HR (95% CI) | p | HR (95% CI) | p |
| **Age**  continuous | 102 (41) | 1.01 (0.98-1.04) | 0.549 |  |  | 105 (49) | 1.03 (1.00-1.07) | **0.041** | 1.09 (1.04-1.15) | **0.001** |
| **Sex**  Female  Male | 43 (17)  59 (24) | 1.06 (0.57-1.98) | 0.845 |  |  | 45 (19)  60 (30) | 1.29 (0.73-2.29) | 0.385 |  |  |
| **Location**  Esophagus  Stomach | 41 (17)  61 (24) | 0.89 (0.48-1.66) | 0.721 |  |  | 41 (19)  64 (30) | 0.95 (0.54-1.69) | 0.871 |  |  |
| **cT stage**  T1-2  T3-4 | 39 (10)  63 (31) | 2.15 (1.05-4.39) | **0.035** | 2.30 (0.84-6.33) | 0.106 | 41 (16)  64 (33) | 1.43 (0.79-2.61) | 0.241 | 1.16 (0.48-2.76) | 0.744 |
| **cN stage**  N0  N1-3 | 60 (21)  42 (20) | 1.44 (0.78-2.65) | 0.245 | 1.66 (0.69-3.96) | 0.257 | 61 (27)  44 (22) | 1.17 (0.67-2.06) | 0.577 | 1.61 (0.77-3.38) | 0.206 |
| **cM stage**  M0  M1 | 94 (37)  8 (4) | 1.15 (0.41-3.23) | 0.790 |  |  | 97 (47)  8 (2) | 0.41 (0.10-1.68) | 0.215 |  |  |
| **Differentiation grade**  Low/Intermediate grade  High grade | 47 (13)  53 (28) | 2.36 (1.22-4.57) | **0.010** |  |  | 48 (15)  55 (33) | 2.33 (1.26-4.29) | **0.007** |  |  |
| **Lauren classification**  Intestinal  Diffuse/Mixed | 55 (18)  46 (23) | 1.76 (0.95-3.26) | 0.074 |  |  | 56 (22)  48 (26) | 1.53 (0.86-2.69) | 0.146 |  |  |
| **Residual tumor status**  R0  R1  R2 | 89 (32)  13 (9)  0 | 3.25 (1.54-6.87) | **0.002** | 2.55 (0.73-8.91) | 0.144 | 89 (35)  14 (12)  2 (2) | 4.28 (2.18-8.42)  13.13 (2.90-59.45) | **<0.001**  **0.001** | 5.23 (1.90-14.39)  14.52 (2.46-85.55) | **0.001**  **0.003** |
| **PODXL expression**  Negative  Positive | 25 (12)  48 (13) | 0.54 (0.25-1.18) | 0.120 | 0.53 (0.22-1.30) | 0.166 | 25 (12)  51 (20) | 0.87 (0.42-1.78) | 0.697 | 1.44 (0.62-3.31) | 0.395 |
| 1) Adjusted for cT, cN, residual tumor status and PODXL expression  2) Adjusted for age, cT, cN, residual tumor status and PODXL expression | | | | | | | | | | |

| **Additional file 5: Table S5b**  **Cox regression**  **Neoadjuvant fluoropyrimidine + oxaliplatin ≥8 weeks of both ± adjuvant chemotherapy NOS, no irinotecan** | | | | | | | | | | |
| --- | --- | --- | --- | --- | --- | --- | --- | --- | --- | --- |
|  | **TTR** | | | | | **OS** | | | | |
|  |  | **Unadjusted** | | **Adjusted^1^** | |  | **Unadjusted** | | **Adjusted^2^** | |
|  | n (events) | HR (95% CI) | p | HR (95% CI) | p | n (events) | HR (95% CI) | p | HR (95% CI) | p |
| **Age**  continuous | 87 (33) | 1.01 (0.97-1.04) | 0.748 |  |  | 88 (36) | 1.02 (0.99-1.06) | 0.212 | 1.10 (1.03-1.16) | **0.003** |
| **Sex**  Female  Male | 35 (11)  52 (22) | 1.52 (0.74-3.14) | 0.255 |  |  | 36 (11)  52 (25) | 1.89 (0.93-3.84) | 0.080 |  |  |
| **Location**  Esophagus  Stomach | 39 (16)  48 (17) | 0.82 (0.41-1.62) | 0.569 |  |  | 39 (17)  49 (19) | 0.81 (0.42-1.57) | 0.537 |  |  |
| **cT stage**  T1-2  T3-4 | 32 (7)  55 (26) | 2.56 (1.11-5.90) | **0.027** | 2.32 (0.75-7.20) | 0.145 | 32 (9)  56 (27) | 2.01 (0.94-4.28) | 0.071 | 1.20 (0.45-3.20) | 0.722 |
| **cN stage**  N0  N1-3 | 50 (15)  37 (18) | 1.80 (0.91-3.57) | 0.094 | 2.34 (0.85-5.90) | 0.104 | 50 (17)  38 (19) | 1.60 (0.83-3.07) | 0.162 | 2.39 (1.00-5.73) | 0.051 |
| **cM stage**  M0  M1 | 80 (29)  7 (4) | 1.46 (0.51-4.17) | 0.476 |  |  | 81 (34)  7 (2) | 0.55 (0.13-2.29) | 0.412 |  |  |
| **Differentiation grade**  Low/Intermediate grade  High grade | 40 (11)  46 (22) | 2.14 (1.04-4.42) | **0.039** |  |  | 40 (10)  47 (26) | 2.67 (1.29-5.54) | **0.008** |  |  |
| **Lauren classification**  Intestinal  Diffuse/Mixed | 47 (15)  40 (18) | 1.60 (0.81-3.18) | 0.179 |  |  | 47 (16)  41 (20) | 1.57 (0.81-3.03) | 0.179 |  |  |
| **Residual tumor status**  R0  R1  R2 | 76 (25)  11 (8)  0 | 4.28 (1.90-9.60) | **<0.001** | 3.41 (0.77-15.06) | 0.105 | 76 (26)  12 (10)  0 | 5.34 (2.50-11.39) | **<0.001** | 4.76 (1.48-15.25) | **0.009** |
| **PODXL expression**  Negative  Positive | 19 (10)  44 (10) | 0.40 (0.17-0.96) | **0.041** | 0.43 (0.15-1.25) | 0.122 | 19 (9)  45 (14) | 0.67 (0.29-1.54) | 0.342 | 1.34 (0.51-3.53) | 0.560 |
| 1) Adjusted for cT, cN, residual tumor status and PODXL expression  2) Adjusted for age, cT, cN, residual tumor status and PODXL expression | | | | | | | | | | |
